# The biological functions of *Lonicera japonica* in animal husbandry and its application in green breeding

**DOI:** 10.3389/fvets.2025.1622768

**Published:** 2026-01-05

**Authors:** Xia Zhang, Hailong Huo, Lijuan Hu, Fuhua Yang, Yunze Deng, Xiaojing Hu, Haizhen Wang, Jinlong Huo

**Affiliations:** 1 Department of Biological and Food Engineering, Lyuliang University, Lvliang, Shanxi, China; 2 Yunnan Open University, Kunming, Yunnan, China; 3 College of Animal Science and Technology, Shanxi Agricultural University, Taiyuan, Shanxi, China; 4 Yunnan Academy of Animal Husbandry and Veterinary Sciences, Kunming, Yunnan, China; 5 College of Animal Science and Technology, Yunnan Agricultural University, Kunming, Yunnan, China

**Keywords:** *Lonicera japonica*, active ingredient, therapeutic potential, biological function, livestock and poultry production

## Abstract

*Lonicera japonica* (honeysuckle) is a traditional Chinese medicinal herb known for its properties of clearing heat, detoxifying, cooling blood, dispersing wind, and exerting anti-inflammatory and antibacterial effects. Research conducted on domestic animals, the bioactive constituents found in *Lonicera japonica* have been demonstrated to potentiate the immune response, thereby augmenting the animal’s disease resistance capabilities. The antioxidant ingredients effectively diminish free radicals within the animal, thereby slowing down cellular aging and enhancing the quality of animal-derived products. The antibacterial and anti-inflammatory properties of its constituents can effectively harmonize the animal’s intestinal microbiota and refine the gastrointestinal milieu. This equilibrium aids in bolstering nutrient uptake and refining the efficiency of feed utilization. Its antiviral components can minimize the presence of drug residues in animal-derived products, thereby enhancing product safety and bolstering market competitiveness. The attributes highlighted demonstrate that the application of *Lonicera japonica* aligns with the principles of green, eco-friendly, and sustainable development. Its usage promotes the advancement of ecological agriculture and the circular economy, fostering a more harmonious and sustainable approach to farming. This paper primarily reviews the key active constituents, therapeutic potential, biological functions of *Lonicera japonica*, and its contemporary applications in livestock and poultry farming. The objective is to offer a comprehensive reference for optimizing the production efficiency and enhancing the product quality of livestock and poultry, as well as for substituting antibiotics with *Lonicera japonica* in animal husbandry.

## 1 Introduction

Antibiotics are widely distributed across nearly all major classes of life, spanning from arthropods and amphibians to reptiles and mammals (1–4). The discovery of penicillin by Alexander Fleming in 1928 represented a groundbreaking milestone in the evolution of antimicrobial drugs (5). Initially, antibiotics were exclusively employed in humans as a critical tool for combating life-threatening diseases (6). During the 1940s, a period that saw an increasing human demand for meat and poultry, extensive research was undertaken in the fields of animal nutrition and feed science to enhance meat production efficiency (7). Antibiotics serve a critical function in preventing, controlling, and treating infectious diseases in both humans and animals. This underscores the importance of using antibiotics in animal feed as a key strategy to enhance feed efficiency, accelerate animal growth, and improve the quality of animal-derived products. From the 1950s to the 1970s, European countries independently established regulatory frameworks to authorize the use of antibiotics in livestock feed. This period coincided with the golden age of antibiotic discovery, characterized by a significant reduction in the cost of commercial antibiotics, which in turn spurred a dramatic increase in demand for antibiotics in agriculture and worldwide (8). Antibiotics, therefore, function as a crucial instrument in facilitating the growth of intensive and large-scale agricultural systems. Nevertheless, the inappropriate use of antibiotics has raised significant concerns regarding the emergence and spread of drug-resistant bacteria (9).

**Figure 1:**
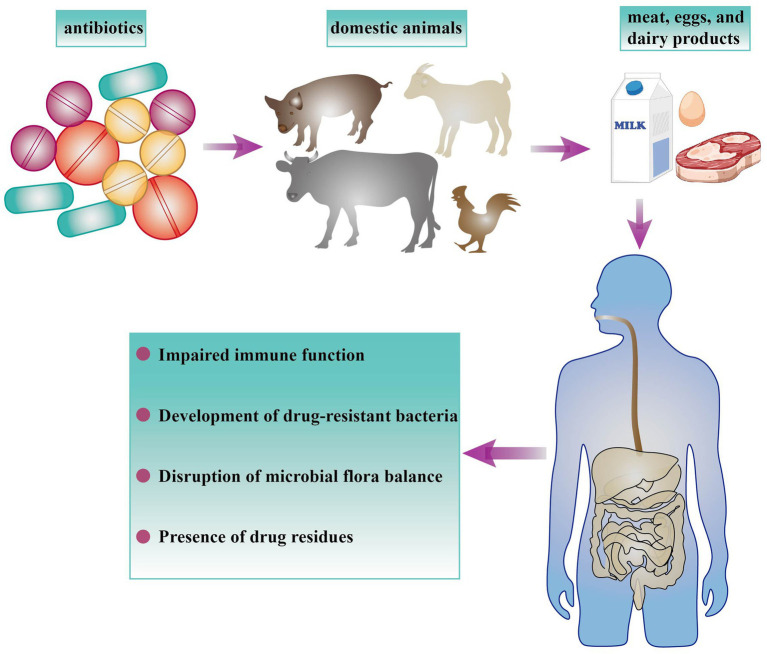
The detrimental impact of antibiotics on human health is a concern. When livestock and poultry are administered feed or medication that includes antibiotics, this practice can lead to a spectrum of potential health implications. Ingesting meat, eggs, and dairy derived from these animals can compromise the immune and endocrine systems, impair liver and kidney function, disrupt the equilibrium of intestinal microbiota, and pose a substantial health hazard to humans.

Research has shown that the growth-enhancing effects of antibiotics are attributable to a decrease in bile salt hydrolase activity, an enzyme produced by gut bacteria that negatively impacts the digestion and utilization of host fats (10, 11). The extensive and often excessive use of antibiotics in livestock and poultry farming has resulted in numerous issues, including the proliferation of drug-resistant strains, diminished animal immunity, the triggering of epidemic diseases, and a substantial reduction in the preventive and therapeutic efficacy of antibiotics. Simultaneously, the substantial presence of antibiotic residues in animal products leads to environmental contamination, disrupts ecological equilibrium, and ultimately re-enters the human body through the food chain, posing significant threats to human health and jeopardizing public health security (12, 13). The following outlines several detrimental effects on human health (Figure 1): a. Decreased immune function. As an illustration, the extended use of antibiotics like tetracycline and oxytetracycline in pig farming can markedly compromise the immune function of pigs, resulting in diminished disease resistance and nonspecific immunity (14). b. Generated antibiotic-resistant bacteria. The extensive use of antibiotics has driven the evolution of pathogenic bacteria into highly resistant superbugs. These resistant strains were released into the environment through animal waste, leading to severe contamination of water and soil, and ultimately posing a threat to human health through the food chain (15, 16). c. Disruption of microbial flora balance. Although antibiotics were effective in preventing and treating bacterial infections, they simultaneously inhibit beneficial probiotics in animals, leading to a disruption of the body’s microbial flora balance. The disruption of the animal’s internal microenvironment facilitates the excessive proliferation of fungi and pathogens, leading to secondary infections and substantially complicating the efforts to prevent and control diseases in livestock and poultry (17). d. Residual drug compounds. The inappropriate utilization of antibiotics in animal husbandry results in the persistence of antibiotic residues within animal-derived foodstuffs, which subsequently were transferred to humans via the food chain. Continued consumption of animal-derived foods containing antibiotic residues is linked to a heightened risk of various chronic health issues in humans (18). The persistence of antibiotic residues within animal products, coupled with the escalating problem of bacterial resistance to antibiotics, represents a pervasive and daunting challenge faced by nations worldwide. In 2010, China, the United States, Brazil, India, and Germany emerged as the leading consumers of antibiotics in food animal production, collectively comprising 23, 13, 9, 3, and 3% of the global consumption, respectively. Projections indicate that by 2030, the respective proportions are anticipated to rise to 30, 10, 8, 4, and 2%, respectively (19). Based on the above, the Chinese herbal medicine *Lonicera japonica* has garnered significant research interest owing to its natural origin, multi-functional properties, and low residual characteristics. Its bioactive compounds demonstrate a range of pharmacological effects, including antibacterial, antiviral, anti-inflammatory, and immunomodulatory activities. In animal husbandry practice, *Lonicera japonica* has exhibited considerable potential as an antibiotic alternative, promoting animal health and enhancing production performance, thereby offering a promising candidate for supporting the development of antibiotic-free farming systems.

## 2 Advantages of Chinese herbal medicine

As the industry evolves, the regulatory oversight of antibiotic residues in livestock and poultry products within our nation has been increasingly rigorous. Antibiotics have undoubtedly resolved numerous challenges. However, they have also introduced certain adverse effects that cannot be overlooked (20, 21). Notice No. 194 from the Ministry of Agriculture and Rural Affairs of China mandates that, effective from July 1, 2020, the use of all growth-promoting drug feed additives, except those derived from Chinese herbal medicine, shall be prohibited. The search for alternatives to antibiotics has emerged as a pressing concern. Consequently, within the realm of animal husbandry, in an effort to fulfill the nutritional requirements of livestock and poultry while enhancing critical economic traits such as growth rate, reproductive efficiency, immune function, and meat quality, researchers globally are diligently refining feed formulations and actively seeking out alternative substances (22–24). Nonetheless, Chinese herbal medicine has emerged as one of the most favored alternatives in this context (25, 26).

**Figure 2:**
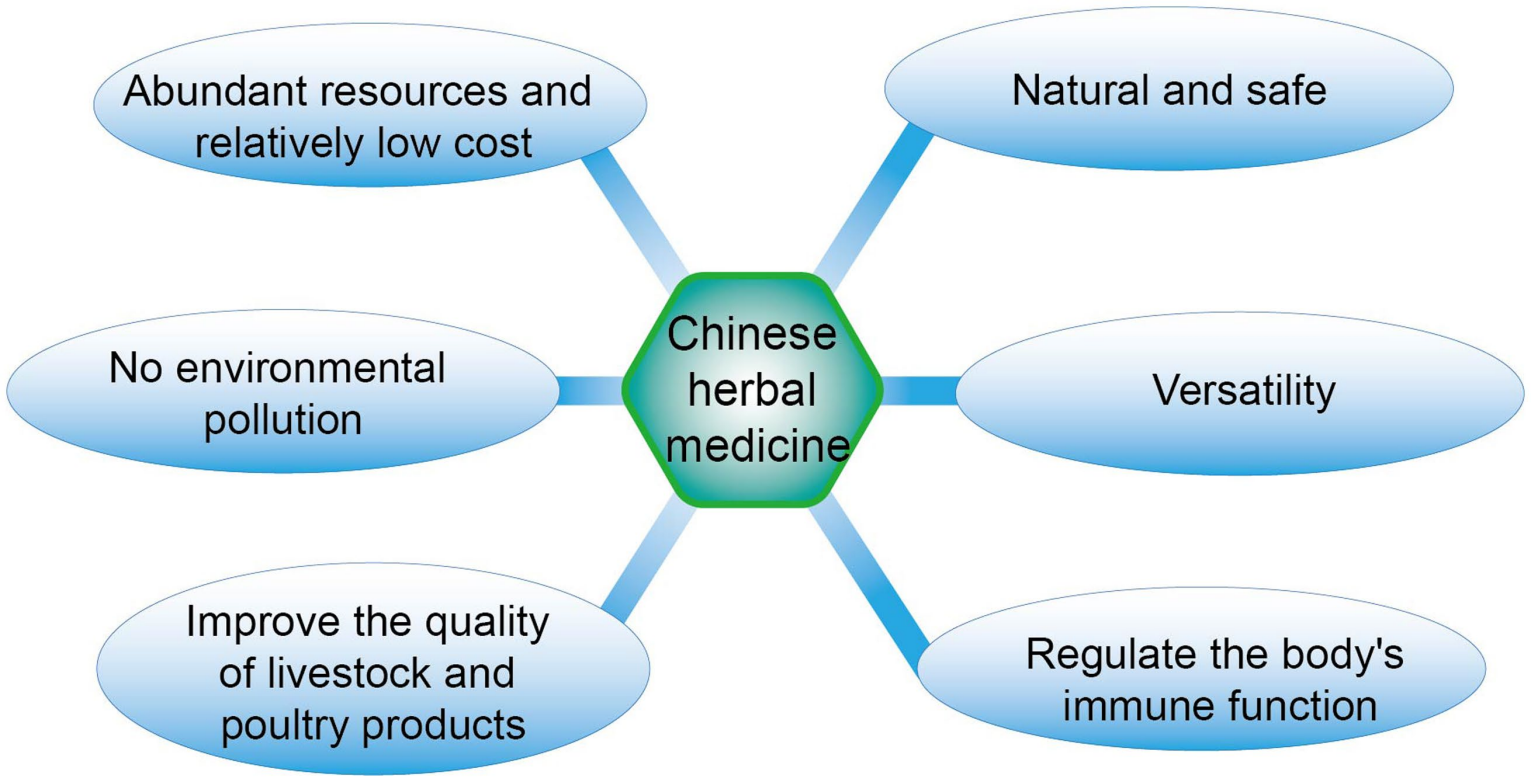
Advantages of Chinese herbal medicine.

Traditional Chinese medicine, derived from a diverse array of natural plant sources, boasts abundant resources, convenience of use, minimal side effects, and a reduced likelihood of residual buildup and the development of drug resistance (27, 28). Consequently, a multitude of traditional Chinese medicinal substances with antibacterial properties, along with their primary active antibacterial components, have become focal points of investigation for an increasing number of scholars (29). Currently, the Chinese herbal medicines that are most frequently employed in animal husbandry encompass Astragalus membranaceus, *Codonopsis pilosula*, Coptis chinensis, Pulsatilla chinensis, *Rheum palmatum*, and Glycyrrhiza uralensis. The primary categories of extracts from these herbs comprise saponins, alkaloids, polysaccharides, terpenoids, tea polyphenols, flavonoids, essential oils, among others (30). These compounds are essential in boosting nutritional levels, mitigating dietary nutrient deficiencies, and enhancing animal digestive functions. The robust safety profile and favorable eco-friendly characteristics of these substances can elevate the umami amino acid content in animal-derived products. Additionally, they can manifest the healthcare benefits of Chinese herbal medicine, thereby significantly facilitating the production of distinctive and premium livestock and poultry products (31) (Figure 2).

## 3 Lonicera japonica

Honeysuckle, scientifically known as *Lonicera japonica*, is a widespread perennial semi-evergreen vine within the Lonicera genus of the Caprifoliaceae family. The flowers of the *Lonicera japonica* vine emerge in clusters at the axils, initially blossoming in a pristine white before transitioning to a golden yellow, which is the basis for its moniker, “Honeysuckle” (32). *Lonicera japonica* is indigenous to China and enjoys a broad distribution across East Asia, including regions such as Japan and the Korean Peninsula (33). This resilient plant boasts a robust adaptability to various growth conditions, withstanding both cold and arid climates, and thrives in sunny environments. It is frequently encountered on slopes, the peripheries of forests, along roadsides, and amidst shrubbery (34). *Lonicera japonica* is rich in a spectrum of functional constituents, such as phenolic acids, flavonoids, saponins, essential oils, organic acids, and more. These constituents endow *Lonicera japonica* with a diverse array of biological activities. The flowers, leaves, and stems of *Lonicera japonica* possess therapeutic properties and are extensively utilized in medicinal applications. Beyond its medicinal worth, *Lonicera japonica* boasts a spectrum of biological functions, including antibacterial, antiviral, anti-inflammatory, antioxidant, immunomodulatory, antipyretic, hepatoprotective, and anti-tumor properties (35). In short, due to its rich chemical components and diverse biological activities, *Lonicera japonica* possesses significant application and research value in the fields of life sciences, medicine, and animal husbandry.

### 3.1 The main active ingredients in *Lonicera japonica*

**Figure 3:**
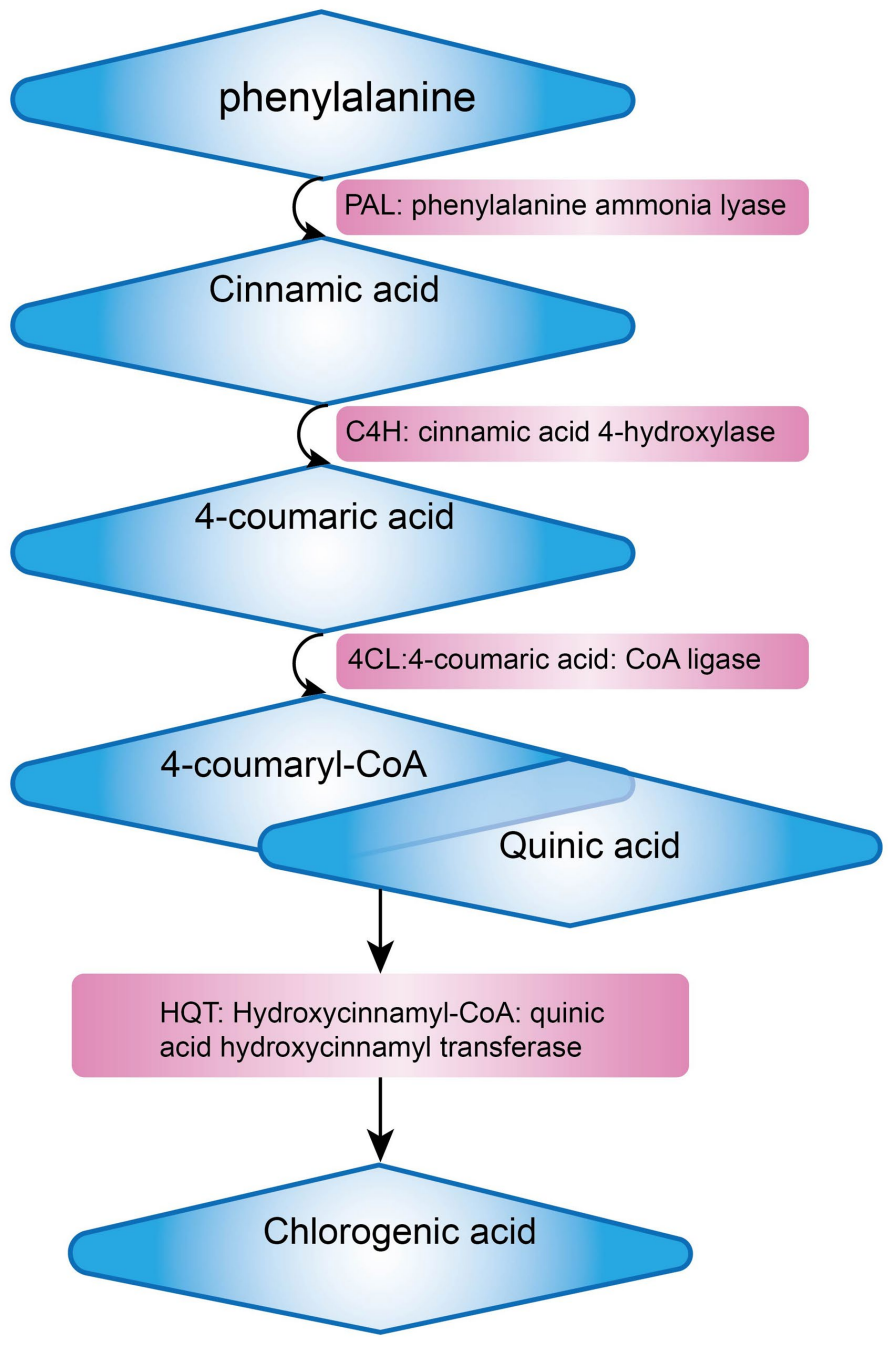
Chlorogenic acid biosynthetic pathway.

*Lonicera japonica* is renowned for its abundance of various bioactive substances, offering a broad spectrum of small-molecule compounds that can be extracted. These compounds, including phenolic acids, constitute a class of secondary metabolites that are universally present in plants, chiefly comprising chlorogenic acid, isochlorogenic acid, and caffeic acid. These compounds possess a diverse array of biological activities and are integral to plant growth, development, as well as in enhancing resistance to diseases, pests, cold stress, and other adverse environmental conditions (36). Chlorogenic acid (CGA) stands out as a pivotal secondary metabolite in plants, primarily synthesized within the cytoplasm and chloroplasts of plant cells. Following its production, CGA is actively transported into the vacuole, where it is stored through specialized transport processes. Its biosynthetic pathway is part of the phenylpropanoid metabolism, with key steps illustrated in Figure 3. Initially, phenylalanine ammonia-lyase (PAL) facilitates the deamination of phenylalanine, resulting in the formation of cinnamic acid. Following this, cinnamic acid undergoes transformation into 4-coumaroyl-CoA via the enzymatic activities of cinnamate 4-hydroxylase (C4H) and 4-coumarate-CoA ligase (4CL). Ultimately, the combination of 4-coumaroyl-CoA and quinic acid is catalyzed by hydroxycinnamoyl-CoA:quinate hydroxycinnamoyl transferase (HQT) to yield chlorogenic acid (37, 38). Chlorogenic acid possesses a variety of pharmacological properties, including anti-inflammatory, antimicrobial, antiviral, anticancer, ultraviolet-resistant, and radiation-protective, immune regulation, lipid-lowering, and blood glucose-lowering effects (39, 40). Isochlorogenic acid, a derivative of chlorogenic acid, possesses a similar chemical structure but demonstrates unique biological activities. In plants, it predominantly occurs as amides, esters, or glycosides, and is seldom present in its free form (41). Caffeic acid demonstrates a range of pharmacological properties, such as enhancing insulin sensitivity, improving glucose metabolism, exerting anti-diabetic effects, and providing liver protection. Research has shown that caffeic acid can alleviate age-related memory loss and reduce the risk of neurodegenerative disorders, including Alzheimer’s disease and Parkinson’s disease (42). These phenolic acid compounds are ubiquitously distributed in nature and play a vital role in the growth and development of plants. Additionally, they serve as valuable medicinal resources and food additives for humans. With the advancement of science and technology, ongoing in-depth research into these compounds will continue to unveil their potential applications in medicine and healthcare.

Flavonoids represent an extensive category of natural compounds prevalent in plant species, boasting a spectrum of biological activities and pharmacological properties (43). Commonly encountered flavonoids include luteolin, quercetin, and rutin. Luteolin, in particular, possesses a broad spectrum of pharmacological activities, such as anti-inflammatory, antitumor, hepatoprotective, and antiviral properties. These attributes were profoundly significant in enhancing the therapeutic quality of *Lonicera japonica* (44). Quercetin exerts a beneficial influence on cardiovascular health by promoting the dilation of coronary blood vessels and enhancing cerebral circulation (45). Rutin is employed to address bleeding issues stemming from capillary fragility and serves as a complementary therapy in the management of hypertension (46).

Saponin compounds represent a significant category of active constituents within *Lonicera japonica*, predominantly comprising triterpenoid saponins alongside other types of saponins. Triterpenoid saponins, complex organic molecules, confer upon *Lonicera japonica* a diverse range of biological activities and pharmacological properties, including immune system enhancement, anti-inflammatory effects, and detoxification capabilities (47). These attributes are pivotal to the application of *Lonicera japonica* in traditional medicinal practices, particularly in the treatment of infectious ailments and in fostering physical recuperation.

Volatile oil is a pivotal active constituent of *Lonicera japonica*, imbued with a diverse array of chemical components and boasting a broad spectrum of pharmacological applications (48). Linalool is a type of monocyclic monoterpene compound that is widely found in various plant essential oils, particularly in *Lonicera japonica*. It imparts a unique aroma to *Lonicera japonica* and also possesses a variety of pharmacological effects, such as antimicrobial, anti-inflammatory, and antioxidant properties (49). Geraniol exhibits a spectrum of biological activities, encompassing antibacterial and anti-inflammatory effects, significantly contributing to the comprehensive efficacy of *Lonicera japonica*. Similarly, eugenol boasts a variety of pharmacological properties, including antibacterial, antioxidant, and analgesic attributes (50).

Malic acid stands out as a crucial organic acid within *Lonicera japonica*, offering a soothing effect on liver damage by mitigating oxidative stress in hepatocytes, thereby supporting the preservation of liver function. In a similar vein, citric acid, another important organic acid, has its primary metabolite as creatine, which plays a vital role in augmenting muscle endurance and facilitating recovery (51).

The polysaccharide constituents found in *Lonicera japonica* encompass both neutral and acidic polysaccharides. These polysaccharide fractions exhibit *in vitro* antioxidant properties, with the acidic sugar moiety showing a higher level of activity. Polysaccharides demonstrate significant anti-inflammatory and anti-tumor attributes in their biological activities. These polysaccharides exert a robust scavenging effect against a range of free radicals, markedly enhancing cellular resistance to oxidation. Furthermore, the polysaccharides derived from *Lonicera japonica* exhibit the capacity to inhibit the proliferation of diverse bacterial and viral strains. Additionally, they suppress the activity of certain inflammatory cytokines and mediators, demonstrating pronounced anti-inflammatory efficacy. *Lonicera japonica* is rich in an array of essential and total amino acids, which are integral to upholding the normal physiological functions within the human body (52). The aforementioned active substances, along with their respective representative compounds, are detailed in Table 1.

**Table 1:** Basic information of main active substances.

Active substance type	Representative substance	Molecular formula	Structure diagram	References
Phenolic acids	Chlorogenic acid; isochlorogenic acid; caffeic acid	C_16_H_18_O_9_; C_25_H_24_O_12_; C_9_H_8_O_4_	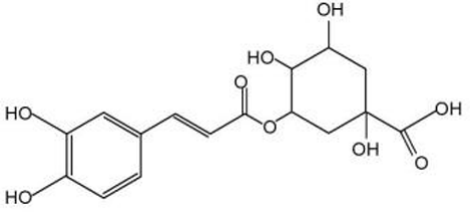 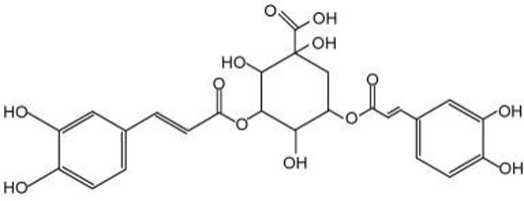 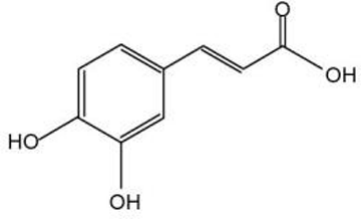	(40–42)
Flavonoid	Luteolin; quercetin; rutin	C_15_H_10_O_7;_ C_27_H_30_O_16_	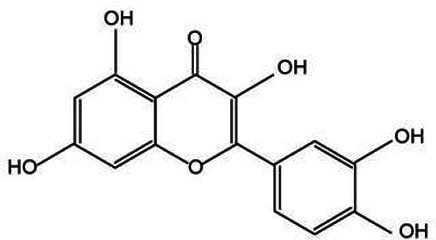 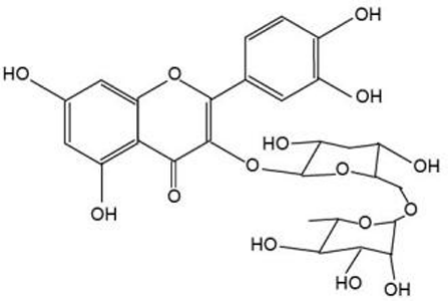	(44–46)
saponins	*Lonicera japonica* saponins		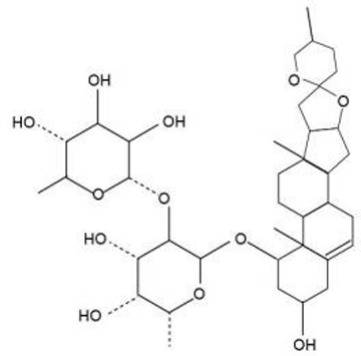	(47)
Essential oil	Linalool; geraniol; eugenol	C_10_H_18_O C_10_H_18_O C_10_H_12_O_2_	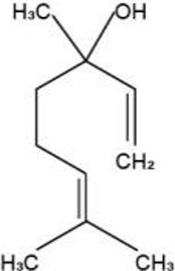 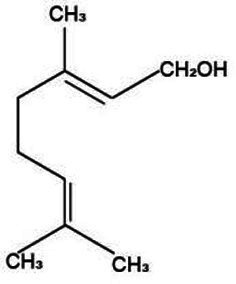 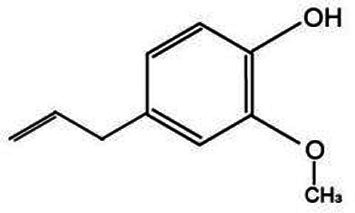	(48–50)
Organic acid	Malic acid; citric acid	C_4_H_6_O_5_ C_6_H_8_O_7_	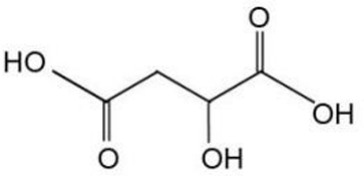 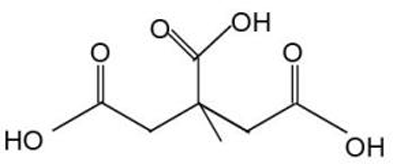	(51)
Iridoids	Loganin Sweroside Secoxyloganin	C_17_H_26_O_10_ C_16_H_22_O_9_ C_17_H_24_O_11_	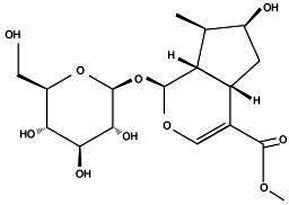 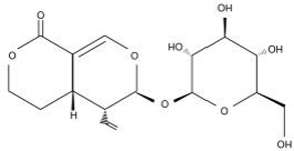 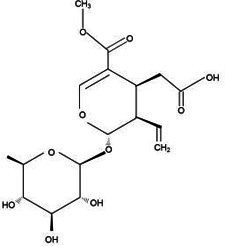	(98–100)
Alkaloids	Stachydrine	C_7_H_13_NO_2_	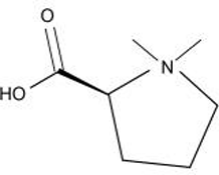	(101)
Others	Polysaccharides; amino acid	(C_6_H_10_O_5_)n RCH(NH_2_)COOH	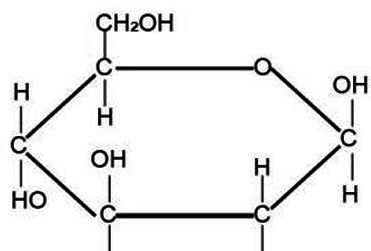	(52)

### 3.2 Medicinal value of main parts of *Lonicera japonica*

*Lonicera japonica* possesses diverse pharmacological properties attributed to its abundant bioactive compounds. The young stems of this plant are characterized by a deep reddish-brown hue and are densely covered with stiff, bristly hairs, glandular trichomes, and fine, soft pubescence, often becoming glabrous near the base. As a semi-evergreen climbing shrub, *Lonicera japonica* demonstrates remarkable adaptability to diverse soil types and climatic conditions, owing to the structural versatility and resilience of its stems (53). Medicinally termed “*Lonicera japonica* vine,” the stem encompasses a variety of bioactive compounds like chlorogenic acid, saponins, and flavonoids. It possesses pharmacological effects including heat-clearing, detoxification, meridian-dredging, pain relief, diuresis, and swelling reduction. Furthermore, notable biological activities are exhibited by the stem, namely tyrosinase inhibition, xanthine oxidase inhibition, and the ability to scavenge nitrite (54).

The leaves of *Lonicera japonica* display a variety of shapes, including oval, oblong-ovate, and ovate-lanceolate. Their wealth of active compounds, including chlorogenic acid, flavonoids, and polysaccharides, accounts for their antibacterial, anti-inflammatory, heat-clearing, and detoxifying capabilities. Moreover, the leaves exhibit hemostatic properties when used externally, along with antioxidant and tyrosinase inhibitory capabilities (55, 56).

The flowers of *Lonicera japonica* exhibit unique features. Typically, they are borne in pairs atop an axillary peduncle, or they may be sessile, forming a whorl at the apex of the branchlets, with each whorl comprising 3 to 6 blossoms (57). The primary active constituents in *Lonicera japonica* include chlorogenic acid, luteolin, and volatile oils, which collectively confer properties such as heat-clearing and detoxification, antibacterial and antiviral action, anti-inflammatory and swelling-reducing effects, as well as potent antioxidant capabilities. The flowers of *Lonicera japonica* are particularly esteemed for their medicinal value. The berries of this plant are generally slender, with a narrow diameter or spherical shape, and are abundant in vitamin C, boasting high antioxidant activity (58). Additionally, the flower buds possess noteworthy anti-cancer and anti-inflammatory attributes.

In essence, the flower of the *Lonicera japonica* plant holds the greatest medicinal worth, brimming with potent active constituents and enjoying the broadest scope of clinical usage, making it the primary medicinal component of the species. The leaves, on the other hand, possess a comparatively lower medicinal value and are predominantly employed topically, often serving as a supplementary therapy for skin inflammations or minor wounds. The therapeutic efficacy of the stem lags behind that of the flower buds; it is primarily utilized to alleviate pain, promote diuresis, and diminish inflammation, making it particularly suitable for the management of rheumatic conditions (Table 2).

**Table 2:** Comparison of medicinal values of main parts.

Position	Main component	Main effect	Primary applications	References
Stem (*Lonicera japonica*)	Chlorogenic acid, saponins, flavonoids	Clearing heat and detoxifying, clearing collaterals and relieving pain, diuresis to reduce swelling	Treatment of rheumatism, arthralgia, joint pain, edema, Soothes itchy skin or sores with topical decoctions	(54)
Leaf	Chlorogenic acid, flavonoids, polysaccharides	Antibacterial and anti-inflammatory, heat clearing and detoxification, external hemostasis	External treatment of mild skin inflammation, mild trauma stop bleeding, soup bath or mash compress the affected area	(55, 56)
Flower	Chlorogenic acid, mignonette glycoside, volatile oil, yellow ketone	Clearing heat and detoxifying, antibacterial and disease-resistant toxic, anti-inflammatory detumescence, anti-oxygen dispel heat	Treatment of wind-heat cold, sore throat, fever, skin infection, clearing heat and relieving summer heat	(57)
Root	Chlorogenic acid, Flavonoids, Volatil oil	Clearing heat and detoxifying, Dispelling wind and activating collaterals, Reducing swelling	Treatment of dysentery, Rheumatoid arthritis, Edema and skin ulcers	(102)

### 3.3 Biological functions of *Lonicera japonica*

**Figure 4:**
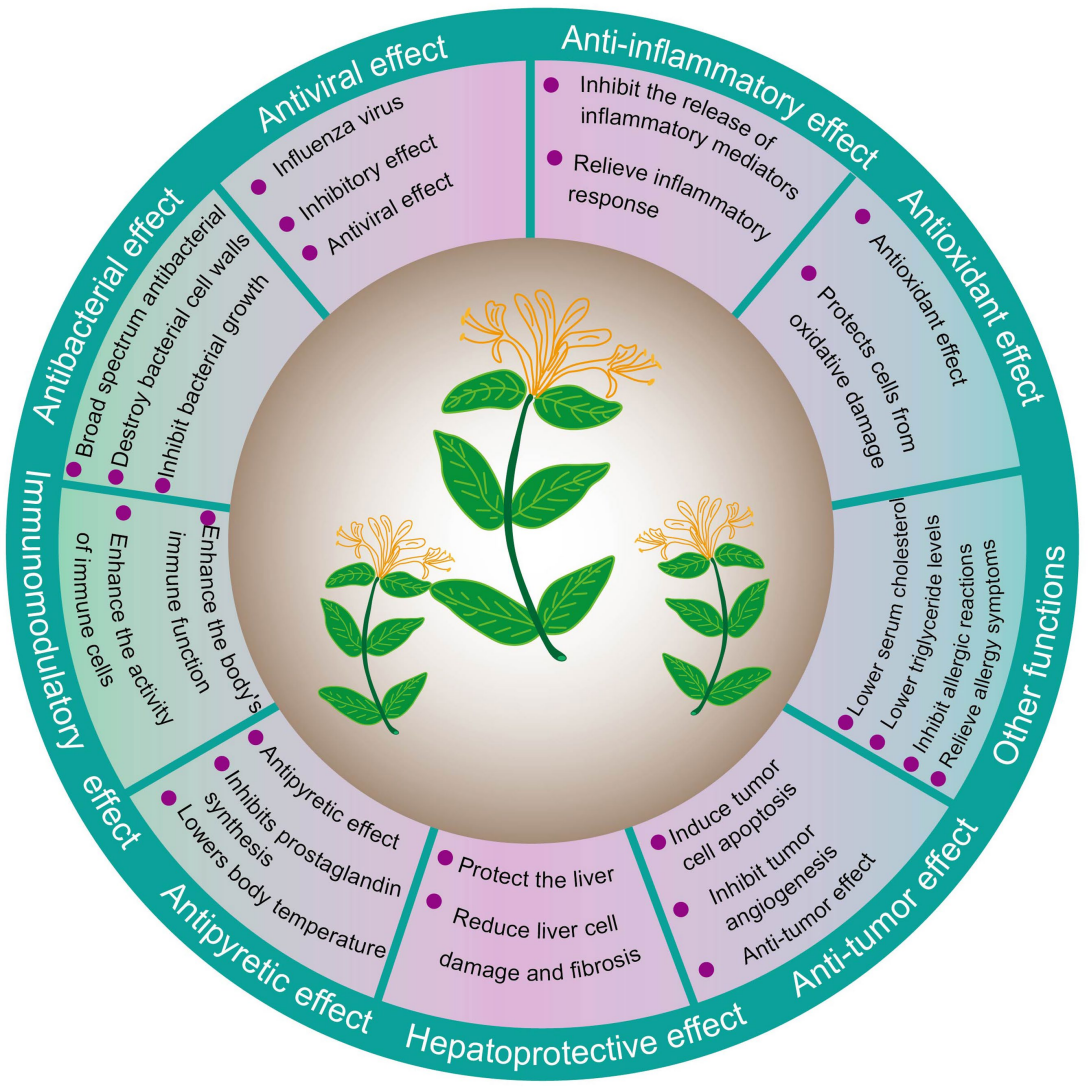
Biological functions of *Lonicera japonica*.

A plethora of active constituents endows *Lonicera japonica* with a diverse array of biological functionalities, including but not limited to antibacterial, antiviral, anti-inflammatory, and antioxidant properties. An exhaustive description of these functionalities is provided below, as illustrated in Figure 4.

(1) *Antibacterial effect*. *Lonicera japonica* displays a strong inhibitory effect on numerous bacterial species, showcasing its broad-spectrum antibacterial capabilities. This includes pathogens such as *Staphylococcus aureus*, Streptococcus species, and *Escherichia coli*. The bioactive compounds contained within *Lonicera japonica*, such as chlorogenic acid and luteolin, are capable of disrupting bacterial cell walls and inhibiting bacterial proliferation (59). A study isolated a new monoterpenoid compound 7-acetyl-8,9-dihydroxythymol and a known 7,8-dihydroxy-9-thymol, extracted from the dried flower buds of *Lonicera japonica*. The objective of the study was to examine the antibacterial properties of these compounds against different bacterial strains, including *Staphylococcus aureus*, *Escherichia coli*, *Micrococcus luteus*, and *Bacillus cereus*. The results indicated that both compounds possessed notable antibacterial effects (60). With *Staphylococcus aureus* and *Escherichia coli* serving as model bacteria, an antibacterial compound screening was performed on extracts derived from *Lonicera japonica* leaves. The leaf extract exhibited potent antibacterial activity, predominantly enriched with 3,5-di-O-caffeoylquinic acid and 4,5-di-O-caffeoylquinic acid. Concurrently, five antibacterial constituents were identified and isolated: 3-O-caffeoylquinic acid, secoxyloganin, luteoloside, 3,5-di-O-caffeoylquinic acid, and 4,5-di-O-caffeoylquinic acid. The antibacterial efficacy of these compounds was ranked as follows: 3,5-di-O-caffeoylquinic acid and 4,5-di-O-caffeoylquinic acid, luteoloside >3-O-caffeoylquinic acid > secoxyloganin. The results showed that these phenolic compounds may have significant antibacterial activity and are also the main reason for the antibacterial activity of *Lonicera japonica* leaves (61). (2) *Antiviral effect*. *Lonicera japonica* possesses a powerful inhibitory influence on a diverse array of viruses, including the influenza virus, herpes virus, and respiratory syncytial virus. It achieves its antiviral efficacy by suppressing viral replication and bolstering the body’s immune response (62). Four distinct extracts of *Lonicera japonica*, including an acid extract, a flavonoid extract, a total extract, and an acid-flavonoid blend, were formulated to ascertain their key antiviral constituents. It was revealed that all four extracts effectively inhibited the replication of influenza strains H1N1, H3N2, and the oseltamivir-resistant variant H1N1-H275Y, with the *Lonicera japonica* acid extract showing the most potent antiviral activity *in vivo*. *Lonicera japonica* extracts demonstrated a broad-spectrum inhibitory effect against influenza viruses, indicating that the acid and flavonoid extracts are the key antiviral components. Furthermore, the acid extract holds promise as a candidate for the development of antiviral pharmaceuticals (63). Investigations have examined the effects of let-7a, induced by *Lonicera japonica*, on the inhibition of enterovirus 71 (EV71) RNA and protein expression, and on viral replication in both cell cultures and live subjects. The data revealed that let-7a had targeted the expected sequence in EV71, causing the suppression of EV71 replication and a decline in viral load. In addition, after lactating mice infected with ev71 were fed *Lonicera japonica* extract or inoculated with let-7a, clinical scores decreased, survival time was prolonged, and viral RNA, protein expression, and viral titers decreased. Ingestion of *Lonicera japonica* attenuated EV71 replication and related pathogenesis *in vitro* and *in vivo*, partially by upregulating let-7a expression. This indicates that *Lonicera japonica* can induce the expression of let-7a, and that this miRNA, as well as 11 other miRNAs, have great potential to prevent and inhibit enterovirus 71 replication (64). (3) *Anti-inflammatory effect*. *Lonicera japonica* possesses the ability to suppress the secretion of inflammatory mediators, thereby diminishing inflammatory reactions. Additionally, it can inhibit the enzymatic activity of cyclooxygenase and lipoxygenase, leading to a decrease in the synthesis of prostaglandins and leukotrienes (65). A study established a sodium dextran sulfate-induced ulcerative colitis mouse model to investigate the anti-inflammatory effect and mechanism of *Lonicera japonica* water extract in the treatment of ulcerative colitis. The findings indicated that all dose groups of *Lonicera japonica* could significantly reduce the levels of IL-6 and TNF-*α*, and the high-dose group had the best inhibitory effect. It led to a significant increase in the population of beneficial bacteria and a decline in harmful bacteria, along with a marked increase in the concentrations of acetic acid, propionic acid, isobutyric acid, valeric acid, and isovaleric acid. Ultimately, it was shown that *Lonicera japonica* can diminish the production of proinflammatory cytokines, increase the concentration of short-chain fatty acids, restore the balance of intestinal ecology, and has a good therapeutic effect (66). An *in vivo* mouse model was employed to study the influence of *Lonicera japonica* on acute pulmonary inflammation caused by lipopolysaccharide (LPS). Female BALB/c mice at 36 weeks of age were injected intratracheally with lipopolysaccharide before treatment with *Lonicera japonica* or vehicle. The ability of *Lonicera japonica* to affect the lipopolysaccharide-induced increase in IL-10 and decrease in TNF-*α*, IL-1β, and IL-6 was tested in the bronchoalveolar lavage of mice. It was demonstrated that *Lonicera japonica* has a protective effect on cytokine release in pulmonary LPS-induced pneumonia, and the anti-inflammatory cytokine IL-10 may be helpful in treating endotoxin-related lung inflammation (67). (4) *Antioxidant effect*. The polyphenolic compounds in *Lonicera japonica* are known for their potent antioxidant effects, capable of neutralizing free radicals, suppressing lipid peroxidation, and defending cells from oxidative stress (68). The antioxidant effect of chlorogenic acid, an extract from *Lonicera japonica*, was studied in relation to the impact of *Mycoplasma gallinarum* infection on the production performance of broilers. The research encompassed an analysis of broiler production performance, serological assessments using slide agglutination, molecular identification through polymerase chain reaction, and histopathological inspections aimed at detecting *Mycoplasma gallinarum*. It was found that chlorogenic acid could increase the live weight of broilers infected with *Mycoplasma gallinarum* and may serve as an alternative treatment option (69). Furthermore, the gastroprotective effect and mechanism of a *Lonicera japonica* water extract, BST-104, were investigated using mouse models of gastritis and peptic ulceration. The results indicated that BST-104 and its active ingredient, chlorogenic acid, exerted antioxidant and anti-inflammatory effects, as well as gastroprotective effects, by downregulating NF-κB expression (70). (5) *Immunomodulatory effect*. *Lonicera japonica* can enhance the body’s immune function, increase the activity of macrophages and lymphocytes, and enhance the activity of immune cells by regulating the secretion of cytokines (such as IL-2 and IFN-*γ*). The study examined the immunomodulatory effect of *Lonicera japonica* stem ethanol extract on Chinese mitten crabs. The total blood cell count and lysozyme activity of crabs fed with *Lonicera japonica* stem ethanol (HSE) diet for 30 days were higher than those of the control group (*p* < 0.05). After infection with *Aeromonas hydrophila*, the treatment with the *Lonicera japonica* stem ethanol extract group enhanced the immune indicators of crabs, including increased hemocyte phagocytosis index and phagocytosis rate, lysozyme activity and nitric oxide concentration (*p* < 0.05). In summary, the *Lonicera japonica* stem ethanol extract has the potential to be developed as a crab feed additive, and can effectively enhance the innate immunity of Chinese mitten crabs after infection with *Aeromonas hydrophila* (71). A series of analyses were undertaken by researchers to investigate the immunomodulatory properties of a water-extracted *Lonicera japonica* polysaccharide on allergic rhinitis. Researchers monitored a range of factors, including behavioral symptoms (rubbing and sneezing), serum inflammatory markers, pathological damage, spleen T cell differentiation, intestinal flora imbalance, and protein expression in the nasal mucosa and colon. The findings revealed a reduction in serum inflammatory factors, eosinophils, goblet cells, NLRP3 inflammasomes, and spleen Th17 cell differentiation in the group treated with water-extracted *Lonicera japonica* polysaccharide. Additionally, the levels of nasal mucosal IL-17 and p-p65, as well as intestinal NLRP3 expression, were decreased. The treatment also preserved the balance of intestinal flora, facilitated the repair of the mechanical barrier, and notably alleviated the behavioral symptoms associated with allergic rhinitis. Allergic rhinitis can be alleviated by restoring the integrity of the intestinal barrier, suppressing NLRP3 inflammasome-mediated inflammation, and modulating the Th17 immune response (72). (6) *Antipyretic effect*. *Lonicera japonica* has a significant antipyretic effect and is often used to treat fever symptoms. It lowers body temperature by inhibiting the synthesis of prostaglandins in the hypothalamic temperature regulation center. In an exploration of the metabolites and metabolomics of *Lonicera japonica* to uncover the pivotal antipyretic active compounds and their modes of action, it was determined that mitogen-activated protein kinase (MAPK) 3 and protein kinase B (AKT) 1 are the key targets chiefly modulated by chlorogenic acid (CA) and swertiamarin (SWE). CA and SWE synergistically inhibited the production of interleukin (IL)-1 and IL-6, alleviated the production of prostaglandin E2, and exerted the same antipyretic effect as *Lonicera japonica* extract at the same dosage content within 3 h (73). (7) *Hepatoprotective effect*. *Lonicera japonica* can protect the liver and reduce liver damage. It reduces liver cell damage and fibrosis through antioxidant and anti-inflammatory effects (74). By exploring the effect of *Lonicera japonica* juice on alcoholic liver disease in mice, it was found that aspartate aminotransferase and alanine aminotransferase in the serum of mice were reduced, indicating that *Lonicera japonica* juice has a hepatoprotective effect. *Lonicera japonica* juice was found to enhance the expression of AMPK, PPARα, and CPT1b in mice afflicted with alcoholic liver disease, effectively diminishing liver lipid content. Additionally, the juice improved the intestinal barrier function in these mice by modulating the expression of mucin and tight junction proteins within the small intestine. It also facilitated the restoration of microbial homeostasis in both the large and small intestines, while boosting the levels of short-chain fatty acids in the cecum. In essence, *Lonicera japonica* mitigates alcoholic liver disease through the reduction of liver and serum lipid accumulation and the regulation of the intestinal microbial-mediated FXR-FGF15 signaling pathway (75). (8) *Anti-tumor effect*. Some components in *Lonicera japonica* have the effect of inhibiting the growth of tumor cells. They exert an anti-tumor effect by inducing apoptosis in tumor cells and inhibiting tumor angiogenesis. The microRNA in *Lonicera japonica* not only plays a physiological role in its original system, but can also be delivered to other species as a potential therapeutic ingredient. Among numerous biological activity studies, miR2911 is an atypical microRNA encoded by *Lonicera japonica*, which has high stability during boiling. The findings indicated that *Lonicera japonica* retarded the progression of colon cancer. miR2911, which can robustly bind to TGF-β1 mRNA, downregulates the expression of TGF-β1 and exhibits high stability even under boiling and acidic conditions. The miR2911 derived from *Lonicera japonica* manifests anti-tumor effects in colon cancer by targeting TGF-β1 mRNA. This downregulation of TGF-β1 facilitates the infiltration of T lymphocytes, thereby impeding the development of colon tumors (76). (9) *Other functions*. *Lonicera japonica* has the capacity to reduce serum cholesterol and triglyceride levels, effectively managing lipid profiles. Additionally, it can suppress allergic reactions, providing relief from associated symptoms.

### 3.4 Adverse reactions and toxicity of *Lonicera japonica*

Although generally regarded as safe, *Lonicera japonica* may cause toxicity when misused, particularly in cases of overdose or prolonged intake. Key concerns include: (1) Gastrointestinal disturbances, most frequently observed in individuals with spleen–stomach deficiency due to its cold nature. (2) Allergic reactions of varying severity induced by specific components. (3) Potential hepatorenal effects, which, despite its usual protective role, require caution at high doses. (4) Elevated risks in vulnerable populations, including pregnant women, children, and patients with autoimmune disorders (Table 3).

**Table 3:** Adverse effects and safety considerations of *Lonicera japonica.*

Adverse reaction types	Main manifestations	Susceptible populations/situations	Prevention and treatment measures	References
Gastrointestinal reactions	Abdominal distension, diarrhea, nausea	Those with spleen and stomach deficiency, overdose	Take after meals and stop when symptoms subside. Avoid long-term and excessive use.	(103)
Allergic reactions	Itching, erythema, urticaria, and severe cases may lead to shock	Those with allergies	Ask about allergy history before taking the medicine, stop taking the medicine immediately and start anti-allergy treatment if mild symptoms occur	(104)
Liver and kidney function effects	Potential risk of drug-induced liver injury	Long-term overdose, those with pre-existing liver and kidney dysfunction	Avoid overdose. Use with caution in patients with liver dysfunction. Long-term use requires monitoring of liver and kidney function.	(74, 105)
Risks in special populations	Potential developmental effects or worsening of symptoms	Pregnant women, breastfeeding women, children, and those with autoimmune diseases	Pregnant women are prohibited from using this product. Other groups should use it after weighing the pros and cons under the guidance of a physician.	(106)

## 4 Application in animal husbandry production

*Lonicera japonica* was widely used in animal husbandry because of its multiple pharmacological effects such as antioxidant, antibacterial, antiviral, antitoxic, antiseptic and anti-inflammatory. With its natural properties of clearing away heat and detoxification, antibacterial and anti-inflammatory, it has become an important natural additive to improve the health level and production performance of livestock and poultry (77–79). It can not only enhance the immunity of livestock and poultry, prevent and treat a variety of infectious diseases, but also improve the digestive function and feed conversion rate of livestock and poultry, thereby improving the overall breeding efficiency. In addition, as a green, residue-free natural medicine, *Lonicera japonica* meets the high standards of modern animal husbandry for food safety and environmental protection, and promotes the sustainable development of animal husbandry. Therefore, the application value of *Lonicera japonica* in animal husbandry cannot be underestimated. It is an important force in promoting healthy and green breeding (Table 4).

**Table 4:** The application of *Lonicera japonica* in animal husbandry production.

Animals	Application areas	Added substances	Application effect	References
Pig	Growth performance	*Lonicera japonica* and Astragalus Blend; Complex herbal extract (including *Lonicera japonica*)	The extract works by improving weight gain, feed utilization, and nutrient digestibility, as well as increasing meat pH and reducing serum cortisol. In weaned piglets, the extract enhanced intestinal health and immune-nutritive function (gut microbiota, villus morphology, reduced diarrhea, and elevated immunoglobulins, cytokines, and serum proteins).	(80, 81)
	Meat quality traits	Chlorogenic acid; *Lonicera japonica* leaf powder	The supplementation improved meat quality by reducing the b-value and increasing inosinic acid content. Dietary supplementation significantly elevated free amino acid content in the serum and longissimus dorsi muscle.	(82, 83)
	Gastrointestinal health	*Lonicera japonica* and *Pueraria lobata* crude extracts; Added chlorogenic acid; *Lonicera japonica*	The extract enhanced growth performance and meat quality by improving average daily gain, feed efficiency, nutrient digestibility, and fatty acid composition, along with immune-antioxidant status. The extract significantly improved intestinal morphology and microbial environment, alongside enhanced blood biochemical and antioxidant profiles. It functions to promote intestinal nutrient absorption and alleviate the associated metabolic load.	(89–91)
Poultry	Growth performance and meat quality	*Lonicera japonica* extract	The extract significantly enhanced broiler health and meat quality, as evidenced by improved blood parameters, reduced spoilage markers in breast meat, and promoted weight gain.	(92)
	Disease prevention and control	*Lonicera japonica*	The additive was effective in increasing body weight and enhancing production performance in broiler chickens infected with *Mycoplasma gallisepticum*.	(69)
	Egg quality	Mulberry leaf, *Lonicera japonica*, and coptis root extract mixture	Dietary supplementation led to a slight improvement in the oxidative stability of eggs.	(93)
	Antioxidant and immunity	*Lonicera japonica* leaves	The treatment significantly enhanced systemic antioxidant status and immune function in broilers.	(94)
	Growth performance and intestinal health	*Lonicera japonica* extract	The supplementation significantly improved growth performance, immune status, and intestinal morphology in broilers.	(95)
Ruminants	Heat stress and immunity	*Lonicera japonica* extract	The extract significantly improved heat stress indicators and boosted immune parameters in dairy cows.	(94)
	Anti-inflammatory and antioxidant	*Lonicera japonica* extract	The extract significantly improved indicators of inflammation, energy metabolism, and oxidative stress.	(96)
	Production performance and digestibility	Chinese herbal complex (including *Lonicera japonica*)	The additive improved post-ruminal digestive enzyme activity, serum antioxidant status, and digestive tract pH.	(97)

### 4.1 Application in pigs

#### 4.1.1 Growth performance

Growth performance is one of the key indicators for assessing the production level in animal husbandry, including aspects such as daily weight gain, feed conversion efficiency, and body weight. Nutritional level is one of the significant factors influencing this indicator, making the study of its impact on growth performance highly important. In a 12-week feed supplement trial, 135 finishing pigs of the (Landrace×Large White) × Duroc breed were studied. The results showed that adding a mixture of *Lonicera japonica* and Astragalus to the feed significantly increased the final body weight and average daily gain during weeks 1– 6 (*p* < 0.05), and also significantly improved the final body weight at the end of the 12-week period (*p* < 0.05). Throughout the trial, the pigs fed with the diet supplemented with the mixture of *Lonicera japonica* and Astragalus showed an overall increase in daily weight gain, an increase in feed utilization efficiency, an increase in the digestibility of dry matter, nitrogen, and energy nutrients, improved meat pH value (*p* < 0.05), and a decrease in serum cortisol concentration (*p* < 0.05). In summary, the addition of the mixture of *Lonicera japonica* and Astragalus can enhance the growth performance and nutrient digestibility of pigs, reduce serum cortisol levels, which is beneficial for improving the meat quality of finishing pigs (80). This study sought to explore the impact of a complex herbal extract formulation—comprising Astragalus, Eucommia, *Lonicera japonica*, and Bitterwood—as a potential alternative to antibiotics on diarrhea incidence, intestinal morphology, nutrient digestibility, and immune function in weaned piglets. A 21-day trial involving 180 weaned piglets was undertaken. The findings revealed that incorporating herbal extracts into the diet notably augmented the populations of Lactobacillus and Bifidobacteria in the piglets’ feces, elevated the height of jejunal villi and the villus height-to-crypt depth ratio, and enhanced levels of immunoglobulin G, immunoglobulin A, immunoglobulin M, interleukin-2, interleukin-4, total protein, albumin, and tumor necrosis factor-*α*. Additionally, the apparent digestibility of energy, crude protein, crude fat, crude fiber, calcium, and phosphorus was significantly improved (*p* < 0.05). The dietary inclusion of herbal extracts also substantially reduced the fecal *Escherichia coli* count and the diarrhea index (*p* < 0.05). In summary, the supplementation of a compound herbal extract preparation in the diet of weaned piglets effectively enhances intestinal morphology, increases feed digestibility, boosts immune function, and lowers the incidence of diarrhea (81).

#### 4.1.2 Meat quality traits

Meat quality traits in animal husbandry refer to the quality characteristics of animal meat products. Characteristics such as color, pH, and texture can determine the taste, nutritional value, appearance, and consumer acceptance of meat products. Feed and nutrition are also important factors affecting meat quality traits. In the livestock industry, chlorogenic acid is considered a potential feed additive that can promote animal health and improve the quality of meat products due to its various biological properties. By supplementing the basic diet with chlorogenic acid, a total of 32 finishing pigs with an average initial body weight of 71.89 ± 0.92 kg (Large White × Landrace) were fed different doses of chlorogenic acid. The results showed that the intermediate dose of chlorogenic acid significantly reduced the meat color b-value and significantly increased the inosinic acid content in the longissimus dorsi and biceps femoris muscles (*p* < 0.01); significantly improved the amino acid composition in the Longissimus dorsi (LD) and Biceps femoris (BF) muscles, and significantly increased the mRNA abundance of Nrf-2, GPX-1, MyoD, MyoG, and the proportion of oxidative muscle fibers in the longissimus dorsi (*p* < 0.05). The results indicated that adding this dose of chlorogenic acid to the diet can promote myogenesis in the longissimus dorsi, induce the transformation of muscle fibers toward a more oxidative type (82). A 60-day feeding trial was undertaken to ascertain the effects of dietary supplementation with Lonicera macranthoides leaf on the growth performance, meat quality, and free amino acid profile of growing-finishing pigs. The findings revealed that the inclusion of Lonicera macranthoides leaf powder in the diet significantly enhanced the free amino acid profile in both the serum and longissimus thoracis muscle of the pigs. Notably, the supplementation led to a substantial increase in the total free amino acid content (*p* < 0.001) and essential free amino acid content (*p* < 0.001) within the longissimus thoracis muscle. These results indicate the potential of Lonicera macranthoides leaf to augment the nutritional value of meat by enriching its free amino acid composition (83).

#### 4.1.3 Gastrointestinal health

The animal intestine, functioning as both a direct communication channel between the internal and external environments and a pivotal defense line for maintaining internal homeostasis, holds self-evident importance (84, 85). Trillions of microorganisms live in the animal’s intestines, forming a complex and sophisticated micro-ecosystem that is vital to the health of animals. First, as the main site of digestion and absorption, it is responsible for converting food into energy and nutrients needed by animals. Secondly, the balance of intestinal microbial communities is essential for maintaining the normal function of the animal’s immune system. At the same time, intestinal microorganisms can also inhibit the growth and reproduction of harmful pathogens by competing for nutrients and living space, thereby protecting animals from infection. Therefore, the health of their intestines is directly related to the animal’s growth, development and overall health (86–88). In animal husbandry, through reasonable feeding management, the use of probiotics and prebiotics and other measures, the balance and stability of the animal’s intestinal microecological system can be maintained, and their disease resistance and production performance can be improved, thereby promoting the sustainable development of animal husbandry.

*Lonicera japonica* possesses potential as a natural antioxidant and beneficial chemopreventive agent, and its extract may offer protective effects against intestinal oxidative damage in animals. An experiment was conducted on 72 triple-hybrid (Duroc × Landrace × Yorkshire) boars, each weighing about 93 ± 2 Kg, which were fed with Chinese herbal additives for 45 days. The findings revealed that the concurrent supplementation of *Lonicera japonica* and kudzu root crude extracts markedly enhanced the average daily weight gain of pigs, while substantially reducing the feed-to-weight ratio, with statistical significance (*p* < 0.05). Oleic acid and monounsaturated fatty acids were significantly increased (*p* < 0.05). There was a significant improvement in blood immune and antioxidant indicators, an increase in the abundance of intestinal microorganisms, and a reduction in potential pathogenicity (*p* < 0.05). There was a significant enhancement in the digestibility of crude protein and total phosphorus (TP) in the feed (*p* < 0.05). These findings indicated that the addition of *Lonicera japonica* and kudzu root crude extracts to fattening pig feed enhanced pork quality, improved serum biochemical indicators, boosted antioxidant and immune capacities, maintained intestinal flora balance, increased the abundance and diversity of probiotics, regulated intestinal metabolic pathways, improved predictions of intestinal microbial phenotypes, and enhanced the digestibility of crude protein and total phosphorus in the feed (89, 90). A study conducted a 28-day trial on 24 weaned piglets at 21 days of age, by supplementing chlorogenic acid in the basal diet to study their intestinal health and regulate selective intestinal flora. The results showed that after adding chlorogenic acid to the diet, the height of the duodenal villi were significantly increased, and the crypt depth was reduced (*p* < 0.05); the height of the jejunal villi and the ratio of the height of the ileal villi to the crypt depth was significantly increased (*p* < 0.05). The blood biochemical indexes were significantly improved, and the antioxidant activity enhanced (*p* < 0.05). In addition, while the number of Lactobacillus in the colon increased, the number of *Escherichia coli* decreased; furthermore, the concentrations of propionic acid and butyric acid in the colon increased significantly (*p* < 0.05). In summary, chlorogenic acid is beneficial for maintaining intestinal morphological integrity and selectively regulating intestinal flora, thus improving intestinal health and growth performance after weaning (90). A study tested the effects of feeding different additives to 21-day-old weaned piglets. These additives included an antibiotic group, a Chinese herbal medicine group (*Lonicera japonica*), and a control group. The results showed that both the antibiotic group and the Chinese herbal medicine group (*Lonicera japonica*) improved intestinal morphology, with the Chinese herbal medicine group (*Lonicera japonica*) also having increased the expression of ileal nutrient transporters (SLC6A9, SLC15A1, and SLC5A1) and having significantly reduced ileal maltase activity and the ratio of small intestine weight to body weight. The addition of the Chinese herbal medicine group (*Lonicera japonica*) had beneficial effects, including regulating intestinal morphology and increasing the mRNA expression of nutrient transporters (91).

### 4.2 Application in poultry

The effects of Saposhnikovia divaricata, *Lonicera japonica*, and *Chelidonium majus* extracts on growth performance, blood cell profile, and meat quality of broiler chickens were investigated by supplementing the basal diet of 240 Arbor Acres broilers aged 1 day. The results showed that white blood cell counts, including neutrophils, lymphocytes, monocytes, and eosinophils in broilers in the *Lonicera japonica* extract group were significantly increased (*p* < 0.05). In addition, erythrocyte counts, hemoglobin levels, and hematocrit values in broilers fed the *Lonicera japonica* extract diet increased, and the volatile alkaline nitrogen and thiobarbituric acid reactive substances values of broiler breast meat decreased. Taken together, suggesting that the inclusion of these plant extracts in broiler diets could potentially improve weight gain, blood cell profile, and meat quality (92). A total of 360 white-feathered broilers with positive mycoplasma serum were selected and divided into an antibiotic group and a *Lonicera japonica* group, respectively. The production performance of the broilers, as well as serological tests, molecular identification, and histopathological examinations, were evaluated with the aim of detecting *Mycoplasma gallisepticum*. The results of the study showed that *Lonicera japonica* not only increased live weight but served as also an alternative treatment option for broiler flocks infected with *Mycoplasma gallisepticum* (69). Three dietary herbal extracts (mulberry leaf: *Lonicera japonica*: coptis root = 48.5:48.5:3.0) were mixed to make a hen feed additive and were fed to 108 28-week-old Roman Brown hens at different concentration levels. The experiment was conducted for 6 weeks, and eggs were collected in the 6th week and stored for 14 days to study the effects of three dietary herbal extracts on egg quality and oxidative stability. The results suggest that dietary administration of three herb extracts may slightly improve the oxidative stability of eggs (93).

The study investigated the effects of Lonicera hypoglauca leaf as a partial substitute in feed on growth performance, serum biochemistry, antioxidant capacity and immune function of geese. A total of 180 one-day-old male swan geese were used in a trial lasting for a 21-day period. It was found that 12% replacement of Lonicera hypoglauca leaf markedly altered the levels of various biochemical parameters in serum such as total protein, total cholesterol, urea, triglycerides, and low-density lipoprotein (*p* < 0.05). Lonicera hypoglauca leaf replacement significantly increased total antioxidant capacity, superoxide dismutase, and glutathione peroxidase activities, while decreasing malondialdehyde content in serum (*p* < 0.05). Furthermore, Lonicera hypoglauca leaf replacement significantly increased the levels of immunoglobulin (Ig)G, IgA, and IgM while decreasing the levels of tumor necrosis factor *α*, interleukin (IL)-1β, IL-2, and IL-6 in serum (*p* < 0.05). In conclusion, partial replacement of feed with Lonicera hypoglauca leaf enhances the antioxidant capacity and immune function of geese without significantly affecting their growth performance (94). A study fed different doses of *Lonicera japonica* extract to 180 28-day-old Holdobaki geese and found that the group fed with the highest dose of *Lonicera japonica* extract had significantly increased average daily weight gain, bursa index, thymus index, and immunoglobulin levels, as well as a significantly increased jejunal and ileal villus height/crypt depth ratio. The analysis of 16 s RNA microbial community diversity demonstrated that the predominant phyla were Firmicutes, Bacteroidetes, Proteobacteria, and Actinobacteria. Notably, there was a significant decline in the abundance of Firmicutes (*p* < 0.01), accompanied by a significant increase in the abundance of Bacteroidetes (*p* < 0.01). The dominant species included Bacteroidetes, Subbacterium variatum, and Faecalibacterium, and the abundance of Bacteroidetes increased significantly (*p* < 0.05) (95).

### 4.3 Application in ruminants

There are limited research reports on the effects of Chinese herbal additives on ruminants, especially *Lonicera japonica* and its extracts. Twenty healthy Chinese Holstein cows with similar levels of milk production, parity and lactation days were selected. The results showed that adding *Lonicera japonica* extract to the diet could significantly reduce the rectal temperature of heat stressed cows (*p* < 0.05). It could significantly reduce the serum creatinine content of heat stressed cows (*p* < 0.05). The serum immunoglobulin G and interleukin-4 levels of heat stressed cows in the medium-dose group were significantly higher than those levels in the control group (*p* < 0.05). In conclusion, adding *Lonicera japonica* extract to the diet may improve the immune response of heat-stressed dairy cows and may help alleviate heat stress in dairy cows (94). A completely randomized design experiment was conducted using 18 Holstein cows with three dietary treatments, in which *Lonicera japonica* extract from 21 days before calving to 30 days after calving. The findings indicated that the extract group significantly diminished the levels of blood biomarkers associated with pro-inflammatory responses (interleukin-1*β*, IL-6, and haptoglobin), energy metabolism (non-esterified fatty acids and β-hydroxybutyrate), and oxidative stress (reactive oxygen metabolites). Concurrently, it enhanced the total antioxidant capacity and superoxide dismutase concentration in the blood. These results indicated that the *Lonicera japonica* extract group had the potential to extend the duration of milk production in dairy cows, augment lactation performance, and bolster the anti-inflammatory and antioxidant capabilities of periparturient dairy cows (96). A study randomly assigned four groups (16 cattle) of cattle to four dietary treatment groups. Through studying the production performance and protease activity of cattle, it was found that the Chinese herbal compound increased the activity of post-rumen digestive enzymes in beef cattle, enhanced the serum antioxidant status, and increased the pH value of the digestive tract. It may be beneficial to the well-being of ruminants (97).

## 5 Future research directions

While *Lonicera japonica* demonstrates considerable promise for application in animal husbandry, its widespread adoption and further development face several challenges that define priorities for future research. Key areas include: (1) *Mechanistic studies*: Compounds such as chlorogenic acid and luteolin exhibit diverse biological activities, yet their precise molecular targets and regulatory mechanisms in animals remain unclear. Modern biotechnologies, such as metabolomics, proteomics, and genomics should be applied to elucidate the molecular basis of its antibacterial, antiviral, immunomodulatory, and meat-quality-enhancing effects. (2) *Standardizing administration*: Optimal dosage, timing, and duration of supplementation vary across species, growth stage, and production goals; scientifically validated guidelines derived from rigorous feeding trials are needed to maximize benefits while minimizing risks. (3) *Long-term safety evaluation*: Systematic assessment of potential tissue residues, reproductive effects, and environmental impacts is essential to confirm its safety as a feed additive. (4) *Synergistic applications*: Investigating interactions between *Lonicera japonica* and other additives (e.g., prebiotics, probiotics, enzymes) or management practices could lead to integrated strategies to further enhance animal health and productivity. Advances in these areas will enable the safe, efficient, and sustainable use of *Lonicera japonica*, contributing to the development of antibiotic-free animal husbandry.

## 6 Conclusion

There are both advantages and challenges in the application of *Lonicera japonica* in domestic animals. *Lonicera japonica* is natural, safe, residue-free, and multifunctional, which conforms to the concept of green breeding. However, the existing problems are that the extraction and stability of active ingredients, standardization of dosage and usage, and safety assessment of long-term use still need further in-depth research. In the future, the mechanism of action of active ingredients can be studied to further clarify the targets of chlorogenic acid, luteolin and other ingredients. We can develop and optimize the types of drugs, develop dosage forms suitable for domestic animals, strive to conduct large-scale application research, and explore the application mode of *Lonicera japonica* in large-scale breeding. In addition, we can evaluate its safety and carry out toxicological research on long-term use. Further promote green and healthy breeding, improve animal welfare, ensure food safety and promote sustainable agricultural development.
